# Bacteria Interactive Cost and Balanced-Compromised Approach to Clustering and Transmission Boundary-Range Cognitive Routing In Mobile Heterogeneous Wireless Sensor Networks

**DOI:** 10.3390/s19040867

**Published:** 2019-02-19

**Authors:** Sercan Yalçın, Ebubekir Erdem

**Affiliations:** Department of Computer Engineering, Firat University, Elazig 23100, Turkey; svancin@firat.edu.tr

**Keywords:** nature(bio)-inspired optimization, wireless sensor networks, cluster head selection, mobile cognitive routing, sink trajectory length

## Abstract

The improvement of stable, energy-efficient mobile-based clustering and routing protocols in wireless sensor networks (WSNs) has become indispensable so as to develop large-scale, versitale, and adaptive applications. Data is gathered more efficiently and the total path length is shortened optimally by means of mobile sink (MS). Two algorithms as bacterial interaction based cluster head (CH) selection and energy and transmission boundary range cognitive routing algorithm with novel approach for heterogeneous mobile networks are proposed in this study. The more reliable and powerful CH selection is made with the greedy approach that is based on the interaction fitness value, energy node degree, and distance to adjacent nodes in a compromised manner. The best trajectories, thanks to intersection edge points of the visited CHs, are obtained in the proposed routing algorithm. In this way, the MS entry to transmission range boundaries of the CH has been a sufficient strategy to collect information. As in energy model, we adopt energy consumption costs of listening and sensing channel as well as transmit and receive costs. Comprehensive performance analyzes have been seriously carried out via the Matlab 2016a environment. We validate that the proposed scheme outperforms existing studies in terms of several performance metrics as simulations.

## 1. Introduction

### 1.1. Background

Through the advances of wireless telecommunications, the development of micro-electronics devices, and implementation of Internet of Things (IoT) in large scales, in the recent decade wireless sensor networks (WSNs) have attracted great attention owing to lots of applications, such as environmental surveillance, smart cities and battlefield, traffic monitoring, tracking medicine and logistic events, military target monitoring [1], health care tracking, and so more [2]. WSNs have handled with distributed sensor nodes in the network on behalf of computational and managament operations for implementing these applications [3,4]. 

In WSN applications, the clustering and routing mechanisms play an important role in energy efficiency and optimization issues [5]. Clustering refers to the division in the nodes in the sensor network into groups, called clusters, according to certain criteria and properties in order to more easily manage the operation of the network [6]. Each cluster has a cluster head (CH), which is selected according to certain algorithms, so that this node can collect data from other normal nodes and process and transmit them to the base station (BS) [7]. In this way, all of the nodes do not need to separately transmit data to BS, and they do not consume energy in vain. CH selection and management are important and difficult problems in clustering. Because, there is C(n,m) combination probability in the selection of m CHs from the n nodes [8]. In addition, passing the data through the relaying nodes while delivering to BS causes this probability to grow. In large-scale networks with very big n values, CH selection probability and the computational complexity increase as an exponential function. In the light of all this information, researchers develop CH selection algorithms that are based on energy efficiency [9,10].

Routing is an important task in the collection of and transmission data to the related locations in WSNs [11,12]. The routing mechanism is the part of the WSNs task that is required to conduct communication setup and data transmission between sensor nodes and CHs or CHs and BS [13]. In order to reduce energy consumption and improve the network life, it is important to develop routing protocols for addressing these problems [14]. Routing protocols are categorized according to many challenges, such as structure of the network, clustering methods, specific features of sensor nodes, node distribution, coverage, and security. Considering these problems for this purpose develop routing techniques. The data that were collected in traditional WSNs are usually transmitted to BS by means of multi-hop logic through specific nodes [15]. Multi-hop communication can provide a certain amount of energy distribution on the nodes, but it leads to a hot spot problem, which means that the energy of the nodes close to the sink node is consumed energy and will hence die earlier [16]. In order to overcome this problem, solutions, such as adapting the node distribution, mitigating the node load, balancing the cluster number, and sink mobility in WSNs are being investigated [12]. Mobility in WSNs brings many advantages that will enable the nodes to more easily transmit data and based on energy efficiency [17]. The data can be dynamically gathered from the respective nodes by means of the mobile sink nodes [18]. Mobile sink nodes collect data by visiting the coverage of nodes, such as CHs that are equipped with specific hardware or software features [19]. Thus, the problem is that the nodes that are remote from the scope of communication cannot directly transmit data to the BS. However, it is a challenging process to determine the motion trajectory of the mobile sink node in WSNs and to determine the least costly paths [20,21]. The aim here is to develop motion paths of mobile sinks that take into account energy, latency, and connection quality. Thanks to these paths, all of the nodes in the WSNs use their energy in a balanced manner and, as a result, the sensor network is able to function for a longer period of time [22,23].

In all of these clustering, routing, and mobility issues, the energy efficiency [24] measures for the WSNs and optimization methods that are more successful in the current WSNs implementations and that may create less fault operations have become the focus of attention of researchers [25]. Optimization methods include functional processes that are inspired by computational intelligence and metaheuristic methods. 

In most engineering problems for science, different techniques and protocols are developed in different fields, such as fuzzy logic, genetic algorithm, and artificial neural networks. The fact that these methods can be adapted, especially in WSNs, clearly shows that novel trends have been introduced in terms of optimization. 

In the field of computer science, different engineering problems and intelligent optimization methods that are based on the concept defined as biological or nature inspired take an important place [8]. In the way of some of the insects or other animals having behaviors, such as moving, searching for food, and interaction with each other based on behavior, such as swarm intelligence; optimization techniques, such as machine automation fault detection, balancing, finding optimal solution, decision making, effective alteration, and comparison with different methods are designed [26].

In WSNs, swarm intelligence and optimization [27] are sub-branches of artificial intelligence techniques that mathematically formulate the behaviors of colonial living animals, such as ants [28], bees, fish, and birds [29,30]. With the structuring of these methods and with metaheauristic approach, innovative solutions are brought to the real world. 

Computer science researchers, therefore, simulate the characters of such animals in the form of swarms to learn the behavior of such animals and find solutions to complex problems [25]. For this purpose, simulations are performed in the same way as ant colony and particle swarm optimization, according to behaviors of insects, such as ants, bees, fireflies [31], or spiders or animals, such as fish, starfish, cat, kangaroo, chicken, elephant, whale, etc [32]. In addition, many researchers propose natural-oriented metaheuristic methods, such as the artificial immune system that was derived from the biological system and functions of humans, digital hormonal system, and bacterial nutrition and foraging optimization [33]. These methods sometimes shed light on NP-hard problems, such as clustering and routing, and sometimes on node distribution in the sensor network [34]. 

WSN development methods [35], such as improving network performance, discarding faulty nodes from the network, reducing energy consumption, or using an optimal number of nodes, can cope with this type of intelligence and nature-inspired optimization approaches [8,26].

Although researchers have developed many clustering, routing, and optimization algorithms, there are still many shortcomings in the mobile WSNs in terms of node allocation, data collection, CH selection, or finding the shortest trajectory length. In existing studies, homogeneous nodes and networks are generally used. However, in the practice of real life, the existence of heterogeneous structures is undeniable. In most studies, CHs that are selected in the first round collect data. However, the CH set to be selected for all rounds and the detection of the location of each CH will facilitate the function of the mobile sink node. In addition, it will be more reasonable to construct paths that occur when MS travels CHs in the shortest manner or by minimizing energy flow and traffic. The optimal complexity of messages that are announced by CHs may be an indication of the designed routing mechanism. In addition, in the energy model, the energy that is consumed as a result of the electronic structure for data transmission and reception, as well as channel listening, data detection, and taking into account the energy consumed in order to avoid collision will be a more realistic solution.

### 1.2. Motivation and Solutions

**Figure 1:**
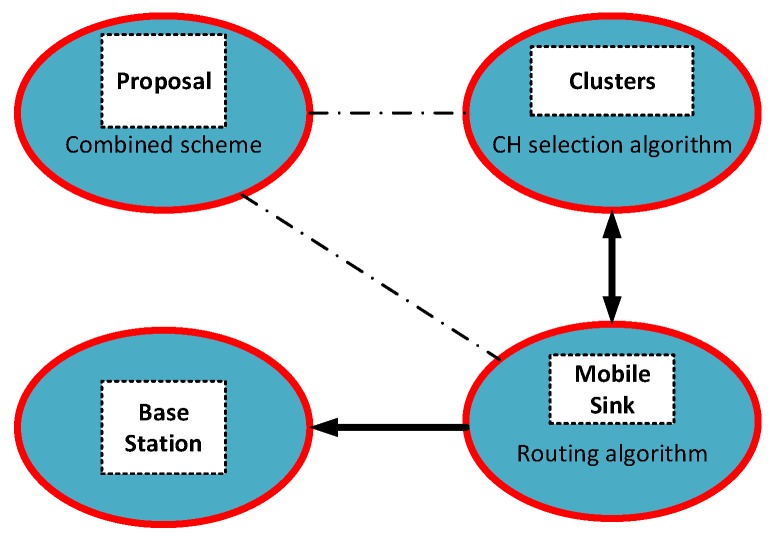
Our proposal design scheme.

This paper proposes a CH-selection algorithm for nature-inspired supervised clustering to provide an innovative approach to mobile WSN applications. This enables the most adaptive CH selection in clustering. We also recommend an energy and transmission boundary range-aware routing protocol that aims to find the best mobile trajectories. The set of CHs that was obtained in the first algorithm is used in the routing algorithm. Figure 1 depicts our proposed combine scheme to the readers.

The proposed scheme and state-of-art studies have been conducted on performance analysis for many control variables and metrics. The performance results clearly emphasize that the proposed methodology has very high performance in terms of network life and energy consumption over improved ant colony algorithm (IACO) in the study [36], particle swarm optimization (PSO) based study that is presented in [37], novel chemical reaction optimization based unequal clustering and routing algorithm (nCRO-UCRA) that is proposed in [38], backbone integrated discrete firefly algorithm based mobile data transporter (BI-DFAMDT) that is presented in [31], and the starfish algorithm proposed in [33] study based bio-inspired and swarm optimization methods.

The major contributions of our study can be summarized, as follows:

- we construct a heterogeneous WSN with mobile sink in order to minimize energy consumption and maximize the network lifetime;
- we develop a CH selection algorithm based on bacterial foraging paradigm. CHs are dynamically selected from the advanced nodes according to the proposed algorithm. These method differs from the existing studies. In this way, we obtain more shorter mobile sink trajectory sets when compared to other works;
- unlike other works, we combine the CH selection algorithm with proposed energy and transmission boundary range-aware routing algorithm developing the mathematically formulations;
- the energy model of our study consists of listening channel and sensing energy consumptions consideration as well as transmission and receiving costs; and,
- in this paper, we have implemented comprehensive simulation analyzes by measuring lots of performance metrics, including average advanced energy usage that we introduced.

The rest of the paper is listed as follows. Section 2 presents the literature survey with state of the art studies. Preliminaries with energy and network model subsections are ordered in Section 3. In Section 4, the proposed algorithms with their methodologies are explained in detail. Section 5 discusses performance analysis results and, consequently, the paper is concluded in Section 6.

## 2. Literature Survey

Many researchers have significantly contributed to studies in the literature. We offer readers the related studies implemented from year 2000 to today in a summary form.

The study [39] proposed a low energy adaptive clustering hierarchy, named LEACH, which selects CHs randomly in the network and provides load balance. However, LEACH-C [40] presents a centralized control algorithm that is improved version of LEACH. In this algorithm, BS knows all of the nodes’ locations and the energy levels are determined in each round.

In a study, Ref. [41] recommends a SEP (Stable election protocol) algorithm, where each sensor node is in a two-level heterogeneous sensor network. The algorithm independently identifies CHs selection according to the first energy relative to the other sensor nodes of the sensor network. 

The study [14] proposes a distributed energy efficient clustering method, called DEEC. In the CH, selection is considered to depend on the rate of the residual energy of the sensor node and the mean energy of the sensor network.

A meta-data based data aggregation scheme is presented for the clustered WSNs [10]. According to this study, CH acquires the data when the sensor nodes sense some events in the transmission range. CH processes the data and then sends it to the BS immediately. However, this operation was considered in a simple manner due to taking much time in the delivery of all data. 

In [42], so as to gather more flexible routes for mobile collectors SenCar, a mobile data collector that is runs like a mobile BS travels the entire network, collects the data from all the nodes, and finally submits the data to the sink via heuristic approach.

The study [17] presents a line-based data dissemination protocol for WSNs using mobile sink. The algorithm is executed according to a line limit value to disperse the data that are acquired in the sense area.

In one study [43], the authors proposed an adaptive algorithm for networks that accommodate the traveling node, while taking into account bacterial mobility. The proposed algorithm is a technique that collects the vector as a linear combination of the last steps of neighboring nodes. This technique allows for bacteria to provide more nutrients. However, in this study, they have only presented a series of mathematical models expressing nutrient distribution for bacteria that have not considered the network model or representing sensor nodes.

In a study [20], an energy-efficient mobile-sink path selection strategy for WSNs is proposed. In the clustering model, a mobile sink travels CHs, named RPs, and then collects data from them. This algorithm provides an energy consumption balance although network delay increases due to the random mobile sink movement.

In [13], authors proposed an energy efficient routing algorithm for improving the network life time. The sink node in the network navigates as hexagon model based multi-hop transmission. The authors consider that the network is divided into some areas and CHs are elected in these areas. Although the algorithm is good at energy balancing, it nevertheless produces communication delay. 

In one study [44], a bacterial foraging optimization based clustering algorithm was proposed. Thanks to the proposed algorithm, energy efficiency along with routing protocol is provided for long tours. However, the proposed algorithm was only compared with the LEACH and [45] PEGASIS protocols.

In [11], a mobile sink with intelligence improved energy efficient model is proposed and then compared with mod-LEACH and PEGASIS protocols.

In [46], authors proposed a novel routing method, called the sleep-wake energy balanced distributed (SEED) clustering method. Thanks to this algorithm, network is split into three energy fields. In this way, CHs in the clusters directly communicate with BS.

In one study [39], the authors proposed the improved ant colony algorithm (I-ACO), which was developed to produce more effective and accurate solutions to the classic traveler salesman problem (TSP). This algorithm combines swarm intelligence and local search manner. With the routing protocol generated, the trajectories were obtained. The proposed algorithm was compared to the existing greedy approach and particle swarm optimization (PSO) models.

In one study [26], the authors presented an energy efficient clustering and routing algorithm with artificial bee colony and metaheuristic approach. They proposed a multi-purpose fitness function using the linear programming formulation for CH selection. As for the routing problem, the transmission path is solved by the cost-based function that is calculated between the number of hops and the energy efficiency. The data in the network is collected in a distributed manner. In this study, routing discovery is designed in a very complex way. At the same way, it can be said that the node and packet load occur because the algorithm is not built on network mobility.

The paper [47] compared the sleep-aware energy efficient protocol named SEED with both the homogeneous and heterogeneous models belong to wireless sensor networks. This algorithm consists of several steps, including setup and CH selection processes. It can be said that these steps do not guarantee the energy balance and consider the packet delivery rate.

In one study [5], CH selection is performed according to its energy level and to the remaining energy. The sensor node with the minimum time delay comparing to others is selected as CH.

In one study [37], the authors proposed a clustering algorithm called EPMS, which was used for WSNs based on the particle swarm optimization-based mobile sink node. In this algorithm, CH selection was performed based on the remaining energy and location of the node. In the routing mechanism, the virtual clustering and mobile sink techniques are combined. The network area was divided into several regions and MS collected the data by visiting CHs. The disadvantages and shortcomings of this study can be listed, as follows. In CH selection, only the remaining energy and position of the node were observed. Node degree, load energy capacity, and fitness value criteria have not been evaluated. The WSN structure is homogeneous. In real life, heterogeneous network models are important. In the energy model, only the energy consumed for transmission and reception is considered. 

In one study, the authors [38] presented a novel unequal clustering and routing algorithm (nCRO-UCRA) that is based on a chemical reaction to address the hot spot problem. The number of elements of the clusters close to the sink node is designed as more than other papers. CH selection was made according to the proposed cost function. As for the routing protocol, it is designed for the chemical and potential energy formulations. However, it can be said that CHs carry data into the sink node with the multi-hop method, increasing energy consumption and the possible packet delay. In one study, authors [31] proposed a bio-inspired decrete firefly algorithm (BI-DFAMDT) with the mobile data transporter (MDT) being represented by the mobile sink node. The trajectory length that was traveled by MDT was kept to a minimum with the nature-inspired metaheuristic approach. The travel time of MDT is minimized in data collection. However, we can say that MDT increases data delivery latency while gathering data from the all nodes as travelling in a non-cluster network. 

A study [48] proposed a routing algorithm that constructs trajectories by MS in order to reduce the energy consumption and delay that are caused by the mobile sink node. In this sense, trajectory models have been designed with different transmission paths with the reduced delay path (RDP) algorithm. MS collect data from specific nodes using these trajectories.

In a study [49], the authors address the joint multi-purpose mitigatation problem by presenting both the clustering and routing problems with link quality base. They introduce a novel individual encoding scheme. Also, the energy consumption model is experienced under the real CC2420 energy radio. The study [33] proposed a routing protocol that consisted of ring canal and radial canal models inspired by the underwater starfish. The radial canal number and ring canal radius have been updated according to the transmission range of the sensor nodes, and the delivery of the data to the target is provided in a single-hop manner. Accordingly, the end to end delay time is reduced. In the network model, homogeneous sensor nodes are used. Since the positions of the nodes are known by GPS, it is thought that this can lead to generate disadvantageous results in energy consumption.

The purpose of study [30] is to evaluate the performance of artificial bee colony optimization algorithm (ABCO). The sensor node with the highest energy in a cluster is selected as CH in a limited period. All field can be re-configure depending on the rest of the CHs.

A study [50] proposed a distributed energy efficiency clustering algorithm in heterogeneous networks, named TBSDEEC. In this study, we formulate the threshold value as balanced and sampled with old value when CH selection in the cluster form. This algorithm outperforms other DEEC variants and the ABCO [30] algorithm in terms of energy efficiency. However, the study lacks network modelling details and data collection issues.

In this study, as stated in the problem definition and existing gaps section, it is necessary to eliminate the deficiencies of the existing studies that are mentioned in this section and to offer adaptive solutions to the WSNs for innovative and real applications. For this purpose, the algorithm framework that we proposed is subjected to performance by using IACO [39], EPMS-PSO [37], nCRO-UCRA [38], BI-DFAMDT [31], and starfish [33] routing algorithms.

## 3. Preliminaries

### 3.1. Energy Model

In energy model, the difference of this study from most energy efficient WSN studies is to consider and analyze the energy consumption of the network in four categories.

- Energy consumed during the transmission process.
- Energy consumed during the receiving process.
- Energy consumed for sensing.
- Energy consumed for listening during the TDMA (Time Division Multiple Access)-cycle.

In previous studies, authors ignored the energy consumption analyzes in sensing and listening processes, because they claimed that the sensor nodes consumed less energy in these processes. However, as our contribution for the energy model, the energy consumption mentioned cases above, as well as the energy that is consumed for transmission and receiving has been taken into account. In this way, more realistic and accurate energy analysis has been done.

#### 3.1.1. Analysis of the Transmission and Receiving Energy Consumption Model 

**Figure 2:**
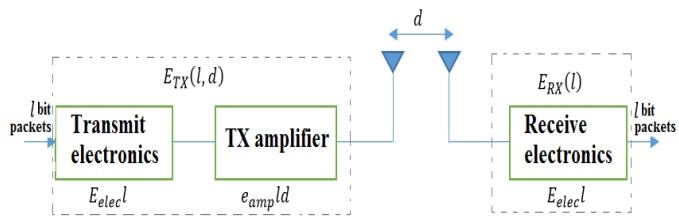
Energy distribution structure.

The energy model of the transmission and receiving energy consumption that is used in this study is based on similar radio model, as used in our previous study [50]. Once it is sent or received, a bit of data to make energy consumption minimize according to distance for this model, the energy consumption of a sensor node for transmission, and processes $E_{TX/RX}$ is measured as Equation (1). Figure 2 illustrates the network energy consumption model. l is data size,$\;E_{elec}$ represents the energy depletion per bit of the node to electronically work the transmission or reception, $e_{fs}$ and $e_{amp}$ indicate the types of radio amplifiers for free space and multiple routes, respectively.$\;E_{TX/RX}\left(l,d\right)$ denotes the energy consumption in sending and receiving data for the l bits.

(1) $$E_{TX/RX}\left(l,d\right)=\{\begin{array}{c} lE_{elec}+le_{fs}d^{2},\;d<d_{0}\\ \;lE_{elec}+le_{amp}d^{4},\;d\geq d_{0} \end{array}$$

We assume that the energy that is depleted for sending and receiving data are the same with each other. The reference distance ($d_{0}$) is computed, as Equation (2).

(2) $$d_{0}=\sqrt{\frac{e_{fs}}{e_{amp}}}$$

In this study, unlike our previous study, the acknowledgement of energy consumption was added to energy consumption. When the data is transmitted to a point, the acknowledgment message that the data is delivered is transmitted. Therefore, the energy that is consumed at this stage should be calculated. For a transmission from a case i to the case i − 1; the average number of desired packet transmissions is calculated, as Equation (3).

(3) $$N_{p}=T_{p}\overset{k}{\underset{i=1}{\sum }}\frac{1}{{\left(1-p\right)}^{i-1}}$$

where $T_{p}$ is the number of packets to transmit, p is the probability value [0,1] of packet transmission, and k is the number of waited transmissions. The number of acknowledgement transmissions can be expressed, as in Equation (4).

(4) $$N_{a}=\frac{T_{p}}{p}$$

In addition, acknowledgement energy consumption cost $E_{ack}$ for a T periyod can be given in Equation (5). Finaly, the total energy consumption $E_{T}$ is calculated, as in Equation (6).

(5) $$\;E_{ack}=\;TN_{p}N_{a}$$

(6) $$E_{T}=E_{TX/RX}\left(l,d\right)+\;E_{ack}$$

#### 3.1.2. Analysis of the Listening and Sensing Energy Consumption Model 

For a listening time t, constant energy is expressed in Equation (7).

(7) $$E_{ce}=A+L_{cost}\cdot t$$

where A is a constant being aware cost and $L_{cost}$ is the listening cost. However, it is necessary to calculate the number of being aware $N_{aw}$ during a TDMA-cycle. Accordingly, the total listening cost in a period T for N sensor nodes, can be given, as Equation (8).

(8) $$E_{list}=\;TN_{aw}\;N\left(E_{ce}\right)$$

We assume that energy consumption during the sensing process is the same as the listening process. In this sense, energy consumption of sensing process $E_{sens}$ can be given in Equation (9).

(9) $$E_{sens}=E_{list}$$

Consequently, the total energy consumption for the above steps can be expressed, as in Equation (10). In this way, we take into consideration these energy consumption formulations in our study.

(10) $$E_{energy-total}=2E_{TX/RX}+2E_{list}$$

### 3.2. Network Model 

**Figure 3:**
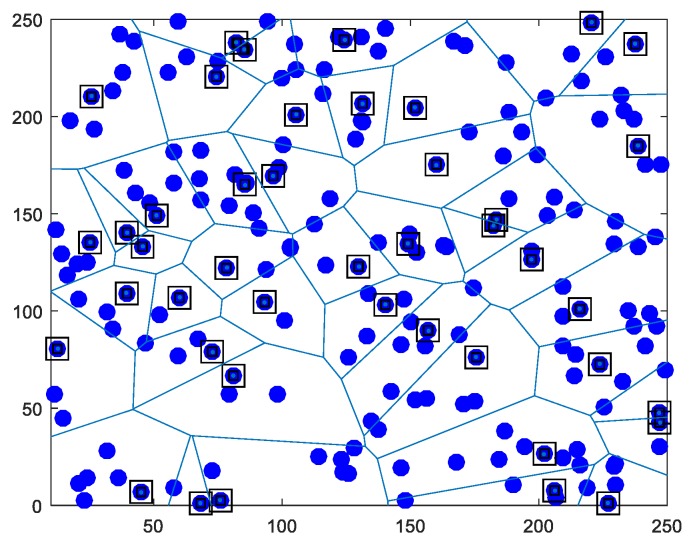
An illustration of the clustered heterogeneous network model in our proposal scheme.

In WSN scenarios in this article, different number of sensor nodes are randomly distributed to the network area in different sizes. The distance of a sensor node from the adjacent nodes can be calculated according to the received signal strength indicator (RSSI) parameter. In this study, mobile heterogeneous distributed clustered WSN was created. The WSN in our study has thre levels of heterogeneous structure, including normal, advanced, and super nodes. In the network, the selected nodes are selected from advanced nodes according to the proposed bacteria foraging based CH selection algorithm. Mobile Sink (MS) node, which collects data from selected CHs, has super node feature. We assume that super nodes have no energy limitation. These different nodes have different properties, such as data processing, communication, and transmission scope. The remaining nodes in the sensor network have the normal node property. Here, it is important to note that, in the proposed algorithm, each CH is an advanced node, but not every advanced node can be assigned a CH. This event is carried out by controlling the energy. Both non-CH and advanced nodes can transfer energy to other normal nodes and provide both a special network model and energy balance. In our study, a different number of sensor nodes were used in the scenarios in order to measure the energy efficiency and network life more accurately. In addition, network size is expressed in numbers in different scales and simulations were performed. Figure 3 shows the proposed clustering formation heterogeneous network model for 200 nodes that were distributed in a 250 × 250 $m^{2}$ network size. In the sensor network, CHs selected from advanced nodes are represented by square-plotted nodes, while others are normal nodes.

## 4. Proposed CH Selection Algorithm Based Bacterial Foraging Paradigm

### 4.1. Overview of the Bacterial Foraging Model

**Figure 4:**
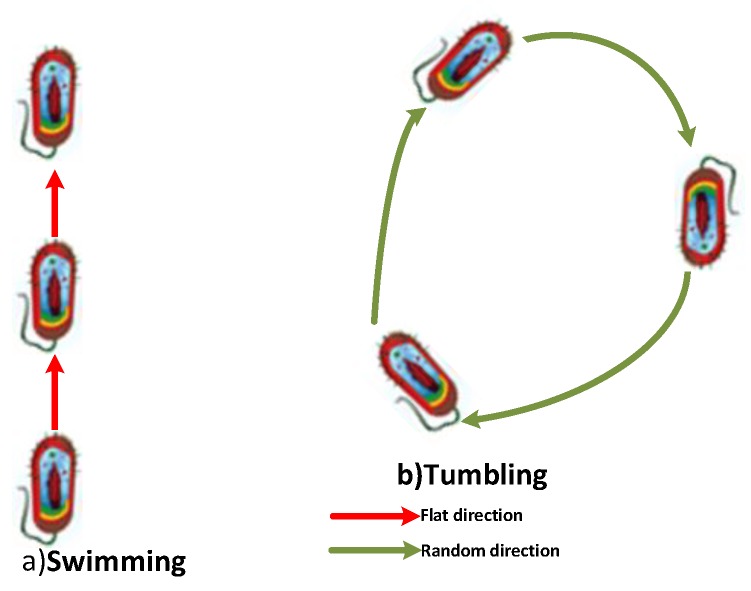
A sample of bacteria chemotaxis movements.

Bacteria are single-cell microorganisms that collect food from the environment in which they are present for certain periods of time in order to survive and provide energy. Escherichia coil and M. Xanthus are examples of these bacteria [43]. In the process of foraging nutrients, the bacteria act as chemotaxis against chemical impulses. In this sense, bacteria have the ability to act differently for specific purposes, called swimming and tumbling. Swimming refers to movement in a direction that is in the direction of locomotive effect, but tumbling refers to different random motion. As seen in Figure 4, it is known that the bacteria move in a flat direction and a fixed path, called swimming, and also move randomly in a unspecified direction, called tumbling, for the search of food at a new point [51].

Bacteria hide their attractive properties and, at the same time, transfer the chemicals to the environment. In this way, bacteria can detect each other in the environment. This effect can be thought of as nodes in the WSNs to transmit messages to each other [52]. Bacteria are found to be active for about one to three seconds to collect nutrients where food is dense. The nutrient density is below a certain threshold and the nutrient is separated from the environment. Given these properties, bacterial movements can be based on the travelling of the sensor node in WSNs. 

In this study, the selection of cluster head (CH) in the heterogeneous cluster network was inspired by the foraging structure of the bacteria [44]. Table 1 presents the similarity and comparison between the bacteria foraging and the WSN.

### 4.2. The Proposed CH Selection Algorithm 

In the proposed CH selection algorithm, a new approach that is based on bacterial foraging belongs to methods, called as swarm optimization, computational intelligence, or metaheuristic, such as swarm optimization, genetic algorithm, ant, and bee colony algorithms is presented. In all proposed algorithms, BC(Bacterial Cell) and BBC(Best Bacterial Cell) represent the sensor node and CH, respectively.

In the proposed algorithm, some of the advanced nodes in the heterogeneous WSN have been assigned to the choice of CH, which can provide the best energy balance of the bacterial cells. In our algorithm, each bacterial cell (BC) was associated with a CH. The best BC can be assigned as CH. With this inspiration, it is possible to identify the best cell in the WSN in the determination of the best cell in terms of energy, degree, and balance load, in order to find bacteria in food and to assign the most appropriate advanced nodes as CH.

#### 4.2.1. Vocabulary

In this sub-section, we describe some notations and their descriptions that were used in the algorithms with Table 2.

The bacteria foraging algorithm consists of three basic steps, including chemotaxis, reproduction, and elimination-dispersal. 

#### 4.2.2. the Step of Chemotaxis

The process in which bacteria move and search for food to increase energy levels is called chemotaxis. Swimming and tumbling are methods of chemotaxis, which describe the movement in two different directions [53,54].

- **Swimming:** In every bacterial cell (BC) action, the bacteria numbered as {1,2,…, i} move and the bacteria {i + 1, i + 2, …, $sn_{num}$} do not move. This manner is much simpler than synchronous decisions regarding swimming to simulate. Algorithm 1 shows the pseudo code of the swimming step.

**Illustration 1:**$\Delta_{m}\left(i\right),m=1,2,\ldots .,p$, is a random number between -1 and 1. Here, the goal is to achieve the result of the swimming movement as seen in line 2 of the Algorithm 1. The counter value in the algorithm is used to calculate the value of the swim length. Line 3 of Algorithm 1 controls that the counter variable does not exceed the maximum swim step. The lines 5, 6, and 7 of the algorithm explain the direction in which the direction of the bacteria moves in the advantageous direction when a direction more advantageous than the previous direction ($J_{previous}$) is detected.

ii. **Tumbling:** In this sub step, each element $\Delta_{m}\left(i\right),m=1,2,\ldots .,p$ produces a random vector $\Delta \left(i\right)\in R^{p}$. We use the formula that is given in Equation (11) for bacterial tumbling movement.
  (11) $$\vartheta^{i}\left(j+1,k,l\right)=\vartheta^{i}\left(j,k,l\right)+C\left(i\right)\frac{\Delta \left(i\right)}{\sqrt{\Delta_{m}\left(i\right)\Delta \left(i\right)}}$$

**Algorithm 1:** An algorithm for the swimming method.1:**Input:**
$sn_{num}$, $N_{s},$
$N_{c}$,$\;N_{re}$,$\;N_{ed}$, $P_{ed}$.2:**Output:** New value of the $\vartheta^{i}\left(j+1,k,l\right)$ after swimming movement.3: counter=0;
/*for computing swim length*/4:  **while**
$counter<N_{s}$
do /*for not exceeding the maximum number of swim steps */5:     counter=counter+1;
6:     **if**
$J\left(i,j+1,k,l\right)<J_{previous}\;$then /* a more advantageous direction*/7:         $J_{previous}=J\left(i,j+1,k,l\right);$
8:         $\vartheta^{i}\left(j+1,k,l\right)=\vartheta^{i}\left(j+1,k,l\right)+C\left(i\right)\frac{\Delta \left(i\right)}{\sqrt{\Delta_{m}\left(i\right)\Delta \left(i\right)}}$
9:     **end if**10:  **end while**11: **return**
$\vartheta^{i}\left(j+1,k,l\right)$


The formulations in these steps represent the chemotaxis movement of the bacteria, where $\vartheta^{i}\left(j,k,l\right)$ represents j th chemotactic, k th reproduction, and l th elimination-dispersal steps in the i th bacterium. C(i) is the size of the randomly dispersal steps and Δ(i) denotes a vector in random direction between its element [−1, 1].

In order to be able to select the appropriate CH in the proposed algorithm, we need to take into account the cost of bacteria in the foraging method, which is inspired. The cost of a bacterium is reduced by its interaction with other bacterial cells. This interaction function $IF\left(sn_{k}\right)$ is calculated in Equation (12), as follows:

(12) $$\begin{array}{c} IF\left(sn_{k}\right)=\overset{sn_{num}}{\underset{i=1}{\sum }}(-d_{attr}\exp(-w_{attr}\overset{P}{\underset{m=1}{\sum }}{\left(sn_{m}^{k}-sn_{m}^{\prime i}\right)}^{2}))\\ +\;\sum_{i=1}^{sn_{num}}(h_{repel}\exp(-w_{repel}\sum_{m=1}^{P}{\left(sn_{m}^{k}-sn_{m}^{\prime i}\right)}^{2})) \end{array}$$

where $sn_{k}$ represents a sensor node (a bacterium cell (BC)), which is the m th component of the k th bacterium and $sn_{m}^{\prime i}$ is the m th component of the k th bacteria. The exponential interaction between these bacteria is taken into account. $d_{attr}$ and $w_{attr}$ are the attractive coefficients and $h_{repel}$ and $w_{repel}$ are the impulse coefficients. These expressions represent the height (effect size) and the width of the impulser, respectively. P is the number of dimensions in a given cell position vector [55,56,57]. 

Algorithm 2 presents the integrated *Chemotaxis()* method that will be used on line 7 of the Algorithm 3. The aim of this algorithm is to update the compatibility value of the bacterium with a random tumbling motion (see 8 th line). 

**Illustration 2:** In the 4th line of the Algorithm 2, the interaction function $IF\left(sn_{k}\right)$ is used to calculate the compatibility value (${\mathrm{BC}}_{fitness}$ fitness value) during the life of the bacterium after the $N_{c\;}$ step. ${\mathrm{BC}}^{\prime },$ and the new BC value is calculated in line 8 and 9 thanks to the *RandomMoveDirection ()* random motion function. In the 14th line of the Algorithm 2, ${\mathrm{BC}}_{health}$ is calculated as an indicator of how many nutrients that BC can acquire during life and how successfully it can avoid the harmful nutrients.

**Algorithm 2:** An algorithm for the chemotaxis step.1:**Input:**
*Population* ($Problem_{size},\;sn_{num},$
$N_{s},$
$Step_{size},\;d_{attr},$
$w_{attr},$
$h_{repel}$,$\;w_{repel})$.2:**Output:** New value of the ${\mathrm{BC}}_{health}$
3: **for**
BC∈
*Population*
and set of advanced nodes
**do**4:     ${\mathrm{BC}}_{fitness}=IF\left(sn_{k}\right)\left(BC\right)+Interaction(BC,$
*Population*,$\;d_{attr},$
$w_{attr},$
$h_{repel}$,$\;w_{repel}$);5:     ${\mathrm{BC}}_{health}={\mathrm{BC}}_{fitness}$;6:     ${\mathrm{BC}}^{\prime }=0;$
7:     **for**$\;i=0\;to\;N_{s}$
**do**8:         *RandomMoveDirection*=*CreateMove*($Problem_{size}$);9:         ${\mathrm{BC}}^{\prime }=$*GetMove*(*RandomMoveDirection*,$\;Step_{size}$);10:         **if**
${\mathrm{BC}}_{fitness}^{\prime }>{\mathrm{BC}}_{fitness}$
**then**11:             $i=N_{s}$;12:         **else**13:             $\mathrm{BC}={\mathrm{BC}}^{\prime }$;14:               ${\mathrm{BC}}_{health}={\mathrm{BC}}_{health}+{\mathrm{BC}}_{fitness}^{\prime }$;15:         **end if**16:     **end for**
17: **end for**18: **return**
${\mathrm{BC}}_{health}$


#### 4.2.3. The Step of Reproduction 

In this step, the first half of the bacterial population survives and the remaining bacteria are located in the same place as their parent and they are divided into two sub-blocks. After the $N_{c}$ steps, the fitness value for ith bacterium is expressed in Equation (13). $J_{health,}^{i}$ is an indication of how many nutrients the bacteria can acquire during their lifetime and how successfully they can avoid harmful nutrients. C(i) is sorted and then compared according to the chemotactic parameter. 

**Illustration 3:** Suppose that, if the ith bacterium that has the highest $J_{health}^{i}$ die, then then the other block, with the other bacteria having the highest $J_{health}^{i}$, being divided into two blocks. Chosen BC Healths are updated according to *Choosen* = *Choose* BC *Health* (*Population*,$\;sn_{num}/2)$ statement. With the reproduction step, the rate of bacteria is kept constant after $N_{c\;}$ steps according to Equation (13).

(13) $$J_{health}^{i}=\overset{N_{c\;}}{\underset{j=1}{\sum }}J\left(i,j,k,l\right)$$

#### 4.2.4. The Step of Elimination and Dispersal 

**Figure 5:**
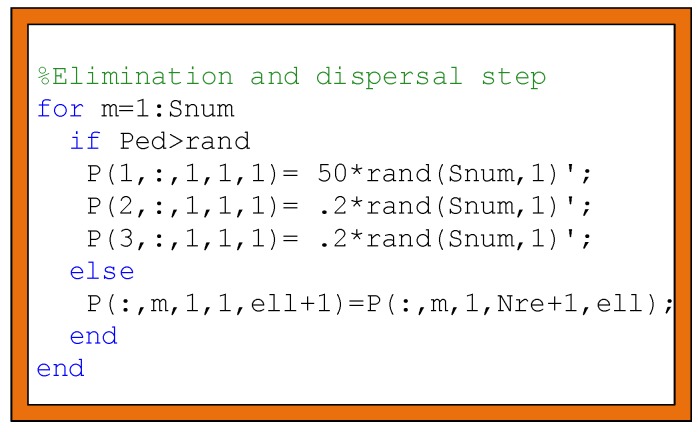
The code block representing the elimination and dispersal step.

The bacterial foraging method removes some bacteria and destroys them. This operation is called elimination and dispersal. $P_{ed}\;\mathrm{each}\;\mathrm{value}\;\in \;\left[-1,1\right]$, which is used after a reproduction event number $N_{re}$, is possible to spread the bacteria in a wide area as a result of the elimination of some bacteria and avoid being trapped in a local area. Figure 5 shows the code block representing the elimination and dispersal step. 

**Illustration 4:** As seen from the Figure 5 for 1 to bacterial cells number ($sn_{num}$), if $P_{ed}$ is bigger than the rand(), for instance, it generated 50 bacterial cells that were distributed population (P) in the sense area. Once this equality is not provided, some bacteria are dispersed according to reproduction event number $N_{re}$ and the elimination number *ell* is used in Figure 5.

In contrast to the existing studies, in the proposed novel CH selection algorithm, the bacterial foraging method based method was used. In this sense, the previously designed algorithm has been developed. In addition, the COST and BandC formulations that are based on the remaining energy, degree, load, and distance to adjacent nodes of the sensor node representing the bacterial cell are considered together.

Unlike other studies, in this study, δ initial percentage is given as the optimal percentage when CH is selected. The δ value does not have a direct effect on the final sets. It only limits the number of CH selection announcements. In this sense, it controls the CH number. The ∝ and β coefficients are used in order to avoid delay by keeping the number of CH that is visited by MS. In this way, energy consumption is considered to be reduced. Each CH has a cost (COST) account and the selection is made according to this criterion. The selection is made based on both the remaining energy of the CHs and the node load. This cost is calculated as in Equation (14), below.

(14) $$COST=\delta \left(\propto \left(\frac{E_{remain}}{E_{max}}\right)+\frac{\left(1-\propto \right)}{\delta }\left(\frac{L_{node}}{L_{max}}\right)\right)$$

**Illustration 5:** Consider, according to Equation (14), that energy consumption is minimized. In addition, the algorithm avoids of sense overlap that is encountered in the network. As seen in Equation (14), the node load is used to prevent transmission delay. The goal is to reduce this value. Due to the fact that only the CH’s transmit data to the MS, this reduces the communication overhead of the WSN. Some nodes can enter the transmission range of CHs. This event is described as sensed overlap. In this case, ties can be discarded between the selected CHs. CH is chosen based on the minimum BandC value that can create a balance and compromise between the node degree and the possible distance around the CH. Equation (15) shows the BandC formulation. By selecting CH that is based on the minimum BandC, both energy consumption and transmission delay are reduced.

(15) $$BandC=\delta \left(\beta D_{node}+\frac{\left(1-\beta \right)}{\delta }D_{dist}\right)$$

**Figure 6:**
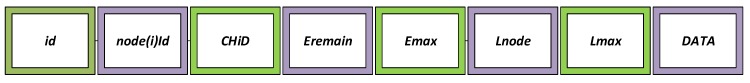
Packet format transferred in the network.

The proposed novel CH selection algorithm that is based on bacterial foraging is presented in Algorithm 3. The aim of this algorithm is to select the best CHs in the network as a result of different movements of bacteria. As shown in line 4, the population is created on the network. In line 8, there is the *Chemotaxis ()* function that is described in Algorithm 2. The COST value that is calculated in Equation (14) is announced to the $N_{neigh}$ that the neighboring nodes of the BCs belong to the population with broadcast message (see line 9, 10). The message packet format is formed as eight segments, including packet *id*, node *id*, CH *id, Eremain, Emax, Lnode, Lmax,* and *DATA,* which is subsequently sent. Figure 6 depicts the packet format that is transferred in the network and the description of the packet segments is given in Table 3. Thanks to this packet, the nodes can compute the COST value. 

**Algorithm 3:** The proposed cluster head (CH) selection algorithm.1: **Input:**
$Problem_{size}$,$\;Step_{size}$, $sn_{num}$, $N_{s}$, $N_{c}$,$\;N_{re}$,$\;N_{ed},$
$d_{attr},\;w_{attr},\;h_{repel},\;w_{repel},\;P_{ed},E_{max},\;L_{max},\propto ,\;\beta ,\delta .R.$ 2: **Output:** BBC,Best Bacterial Cell.3: *Compute the*
COST and BandC values according to Equations (14) and (15), respectively;4: *Population*=*InitializePopulation* ($sn_{num}$,$\;Problem_{size}$);5: **for**
l =0 to
$N_{ed}$
**do**6:     **for**
$k=0\;to\;N_{re}$
**do**7:         **for**
$j=0\;to\;N_{c}$
**do**8:             *Chemotaxis*($Problem_{size},\;sn_{num},$
$N_{s},$
$Step_{size},\;d_{attr},$
$w_{attr},$
$h_{repel}$,$\;w_{repel}$);9:             **for**
BC
∈
*Population*
**do**10: Broadcast the COST value as packet format defined in Figure 6.11:   **if**
$IF\left(sn_{k}\right)\left(\mathrm{BC}\right)\leq IF\left(sn_{k}\right)\left(\mathrm{BBC}\right)$
and obtained COST≤COST
**then**12:               BBC=BC; //save the BC set of BBC
13:**else**
14:BC will be assigned to BBC cluster with minimum BandC
within transmission range R;15:                 **end if**16:             **end for**17:         **end for**18:         *OrderBCHealth(Population)*;19:         *Choosen*= *Choose* BC
*Health* (*Population*,$\;sn_{num}/2)$;20:         *Population= Choosen*;21:     **end for**22:     **for**
BC∈
*Population*
**do**23:         **if**
$Rand()\leq P_{ed}$
**then**24:             BC=*Create*BC*InRandomPosition*();25:         **end if**26:       **end for**27: **end for**28: **return**
(BBC);

**Illustration 6**: If the $IF\left(sn_{k}\right)\left(\mathrm{BC}\right)$ interaction function is smaller than $IF\left(sn_{k}\right)\left(\mathrm{BBC}\right)$, and if the obtained COST is less than the COST, then the new BBC will be found. Otherwise, the BC will be assigned to the BBC with the minimum BandC in the transmission range R. 

On the 18th to 20th lines, that ${\mathrm{BC}}_{health}$ values are listed based on the number of bacteria divided by the reproduction step (see 19 th line). On lines 22th to 24 th of the Algorithm 3, the new position of the BC is randomly assigned using the *CreateBCInRandomPosition ()* function if the number 0 to 1 produced by rand () in the BCs that were included in the population is less than $P_{ed}$. Finally, in the 28th line, the most appropriate BBCs have been found.

As opposed to exsisting other studies [58], many selection parameters are used with the proposed CH selection algorithm. In this way, the maximum energy efficiency is targeted. In addition, the heterogeneous WSN feature provides the intention to be selected from the advanced nodes of the CHs (see line 1 of Algorithm 2).

### 4.3. Proposed Energy-Transmission Boundary Range-Cognitive Routing Algorithm

**Figure 7:**
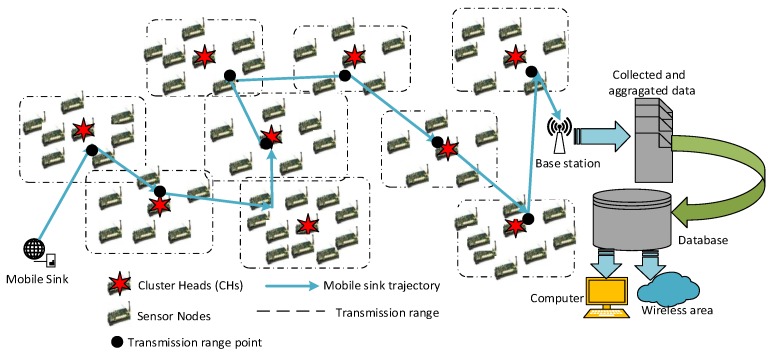
A sample of Mobile Sink (MS) trajectory.

The proposed transmission-aware single hop to MS algorithm is a trajectory algorithm that is generated by selected visiting CHs by MS based on the proposed CH selection algorithm in Section 4.2. The objective of this algorithm is that the MS can navigate all of the CH nodes and collect the necessary information. The collected data is transmitted to BS. An exemplary MS trajectory showing all of these processes is shown in Figure 7.

**Figure 8:**
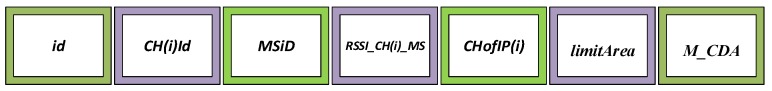
The routing packet format transferred in the network.

The important aspect of this proposed algorithm is to reduce the tour time and the length that MS travels. In this sense, the penetration of the MS into the transmission range of the CHs will be sufficient to acquire data, as seen transmission range points from Figure 7. This means that it does not need to enter the exact position of the CH in the clusters that have different sensor node numbers. Accordingly, in a delay-aware method, the MS trajectory will occur. Afterwards, MS delivers all of the collected and aggragated data (*M_CDA*) to the base station (BS). Thanks to BS, the data are saved as data or information to databases. Finally, someone who wants to view or use the data can accsess them from the computer or wireless channel. The pseudo-code of the proposed algorithm is provided in Algorithm 4. For route discovery CH(i) to MS, the routing packet format that is transferred in the network can be given in Figure 8 and a description of the routing packet segments is given in Table 4.

**Algorithm 4:** Proposed energy-delay and transmission aware routing algorithm.1:**Input:** Set of CH [$CH_{1},CH_{2},\ldots .,CH_{n}].$
2:**Output:** trajectory of MS.3:*Compute the path according to cruscal method*;4:*trajectory=cruscal path*;5:*limitArea*=set of trajectory edges;6:**for**
i=1 to n **do**
7:   x =0;8:   Dist=*calculate* RSSI distance between $CH_{i}$ and MS;9:   **if**
Dist≤ R(i)
**then**10:       x =1;11:       *limitArea=limitArea of trajectory*;12:       **end if**13:   **if**
x =1 **then**14:       **if**
IP≠∅
**then**15:           CHofIP(i)=set of the closest IP for R(i);16:       **end if**17:       **if**
CHofIP(i)=∅
**then**18:             IP=IP and CHofIP(i);
19:       **end if**20: **end for**

**Figure 9:**
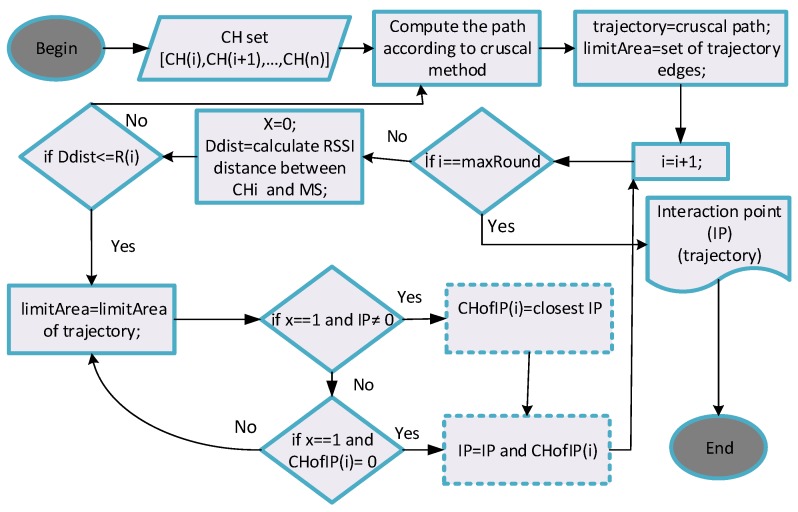
A flowchart of MS trajectory discovery.

**Illustration 7:** Intersection points are updated as IP=IP and CHofIP(i). Hence, d the best MS trajectories are obtained on behalf of energy aware transmission routing mechanism. According to Algorithm 4, the input values as a parameter are a set of CHs. The output is the trajectory belonging to MS (line 1–2). First, starting from the first CH, when considering the MS, a path is created with the cruscal method, which is one of the minimum spanning tree algorithms. This path is assigned to the *trajectory* (line 3–4). As seen on line 5 of the Algorithm 4, trajectory edges are recorded in the edge section called *limitArea*. For the CH number, the first x value is 0. This x value in the boolean data type determines whether the MS is in the CH transmission range or not. Dist distance between CH and MS is measured (line 6-8). If this distance is less than or equal to the transmission range R(i), then the x value is set to 1 and the trajectory boundary edge is assigned to the previously defined *limitArea* (line 9–11). These boundary edges describe the formation of a trajectory that may be sufficient by the joining of MS into the transmission range of the CH. This prevents delays resulting from navigating (delay-aware effect). Subsequently, if the value of x is 1, then the nearest called *intersection point* (IP) is assigned to the cluster (CHofIP(i)), called the intersection point, which intersects the transmission scope in the edge zone (line 13–15). If CHofIP(i) is the empty set, then CHofIP(i) is included in IP and the cluster is merged (line 17–18). Thus, a trajectory belonging to the MS will be found. Figure 9 presents a flowchart that explains the discovery of the MS trajectory.

## 5. Simulation Results

### 5.1. Simulation Setup

This study, together with the proposed algorithms, was performed using the Matlab 2016a program, which runs on an Intel core i7 processor with a 3.40 GHz CPU and 8GB of RAM. Experimental results were obtained according to many network and simulation parameters. The simulation results were carried out on the basis of network dimensions of 250 × 250 $m^{2}$ and 500 × 500 $m^{2}$ represent scenario 1 and scenario 2, respectively. The rest of the parameters that were used in the scenarios are same with each other. In order to measure the sensor node density, analyzes were performed using a randomly distributed 200 to 1000 sensor nodes in the detection area. Two network scenarios that were used with these two network dimensions are considered. In the network model, heterogeneous network structure is designed as normal, advanced, and super nodes. Advanced nodes were used for the selection of CH. The MS node is designed as a super node without energy restriction. One-fifth of the total number of nodes is distributed as advanced nodes with two times the energy from normal nodes. The transmission ranges are 50 m and 80 m for normal nodes and advanced nodes, respectively. In the proposed bacterial foraging-based CH selection algorithm, 50 to 100 bacterial cells were distributed in the initial population, but the proposed algorithm showed better solutions when using 72 bacterial cells. The proposed algorithm was performed 100 times for 0.5 h simulation time. The average of sample data that were obtained to assess network and energy performances was taken into account in the graphs. Table 5 and Table 6 provide a list of the network and proposed algorithm parameters that were used in the simulations. All of the algorithms used in simulations were tested under the same comparison conditions.

**Figure 10:**
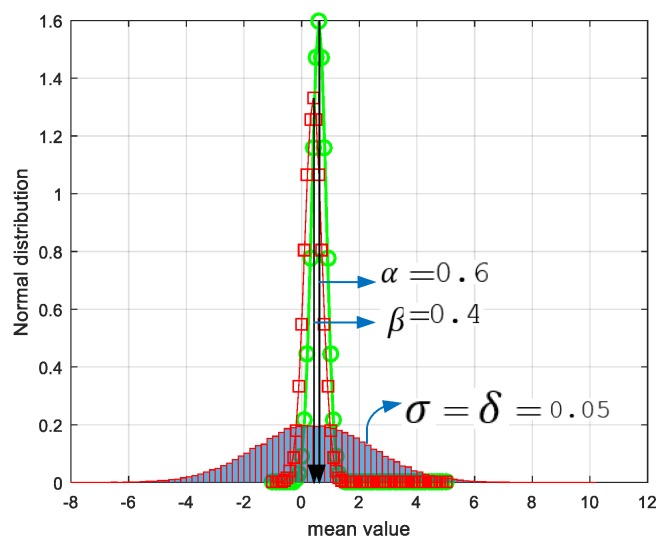
Determination of the ∝, β, and δ values according to normal distribution function.

The possibility intensity function for a normal distribution with mean μ, standard deviation σ, and variance $\sigma^{2}$ is given in Equation (16). ∝ is used for the trade-off between residual energy and node load. β is used for the trade-off between load distribution and energy consumption. As seen in Figure 10, after some simulation iterations, we set ∝ and β at 0.6 and 0.4, respectively. 

We found these values in appropriate mean form, according to normal distribution. Additionally, we assume that δ is the same value as that of σ is 0.05 in the simulations.

(16) $$f\left(x,\mu ,\sigma \right)=\frac{1}{\sigma \sqrt{2\pi }}\exp\left[-\frac{{\left(x-\mu \right)}^{2}}{2\sigma^{2}}\right]$$

### 5.2. Performance Evaluation

To evaluate our study versus existing algorithms, the simulations were caried out in terms of performance metrics with their results being presented below.

**Figure 11:**
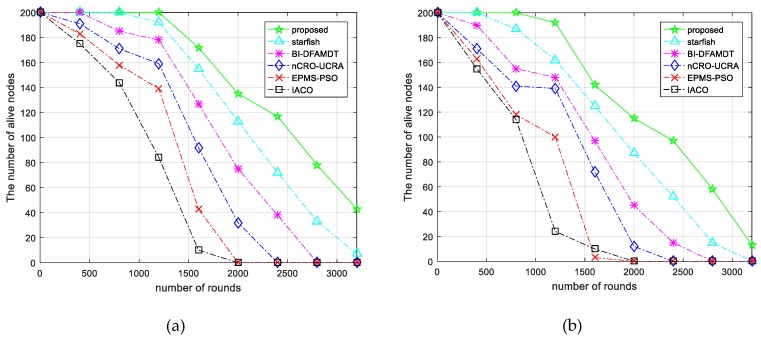
Comparison of the proposed scheme with existing algorithms versus number of alive nodes in the network: (**a**) Simulation graph for scenario 1; and, (**b**) Simulation graph for scenario 2.

- Number of alive nodes in the network: The alive nodes metric is taking into consideration the first node’s starting to die and all the nodes’s dying. We have tested our proposed algorithm over the other protocols varying two scenarios, including scenario 1 and scenario 2 in terms of the number of alive nodes in the network. We have used 200 nodes that are distributed in the network. As seen in Figure 11a, the nodes starts to die after the 1224 th round, while using our algorithm in the scenario 1. This result validates that the proposed method outperforms the existing algorithms for longer rounds. This is because the proposed algorithm is able to utilize the network energy in a balanced manner. Additionally, as shown in Figure 11b, all of the algorithms provide less performance than before when we simulate them in scenario 2. However, our algorithm is still the best performance in comparing the other algorithms. We notice that it requires more sensor nodes deployed in the sensing area to continue for longer rounds in large-scale wireless sensor networks.
- Total remaining energy of the network: By this performance metric, the total residual energy over the lifetime of the network is calculated. We have comprehensively simulated the performance of the proposed algorithm over the other algorithms in both scenarios in terms of the remaining energy of the network versus the number of rounds.

**Figure 12:**
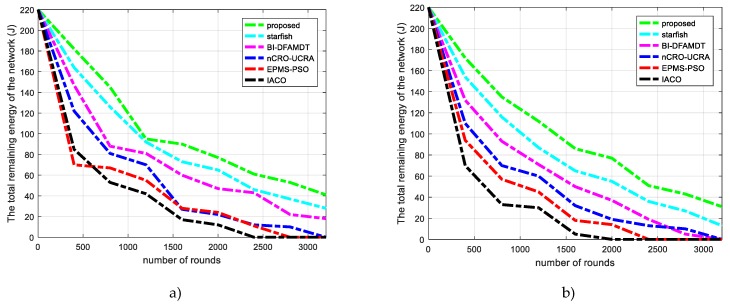
Comparison of the proposed scheme with existing algorithms versus total remaining energy of the network: (**a**) Simulation graph for scenario 1; and, (**b**) Simulation graph for scenario 2.

**Figure 13:**
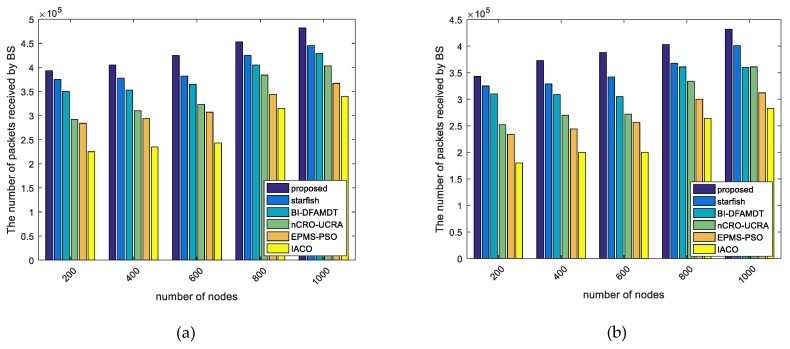
Comparison of the proposed scheme with existing algorithms versus number of packets received by BS: (**a**) Simulation graph for scenario 1; and, (**b**) Simulation graph for scenario 2.

We have used 200 nodes that were distributed in the network. It can be observed that, when using our proposed methods, the total energy of the network is completely consumed after longer rounds when compared with the existing protocols. For example, as shown in Figure 12a,b, the remaining energy of the proposed algorithm in 3200th round is the highest value with approximately 40 J and 17 J in scenario 1 and scenario 2, respectively. It can be said that the nodes use up energy of the network rapidly in the large-scale networks.

iii. Number of packets received by BS: By this performance metric, the total number of packets received by BS is taken into account. We have run the algorithms with respect to the number of packets received by BS varying from 200 to 1000 sensor nodes for scenario 1 and scenario 2. As seen in Figure 13a, if our proposal protocol is used in the simulations, the total number of packets that are received by BS is almost 3.9 × $10^{5}$, 4.1 × $10^{5}$, 4.25 × $10^{5}$, 4.5 × $10^{5}$, and 4.8 × $10^{5}$ for 200, 400, 600, 800, and 1000 nodes, respectively. According to Figure 13b, the total number of packets received by BS considerably diminishes because of more packet losing in the scenario 2 than another. We observe the most and least data packets obtained by the BS in the proposed method and I-ACO. This is due to the fact that our algorithm developed as combine and mobile structure, and can be delivered the packets to the destination more accurately when compared to others.
iv. Trajectory length travelled by MS: This is total routing path length visited by MS. We have experimented all of the algorithms according to trajectory length performance parameter varying from 200 to 1000 sensor nodes that were deployed randomly in the network.

**Figure 14:**
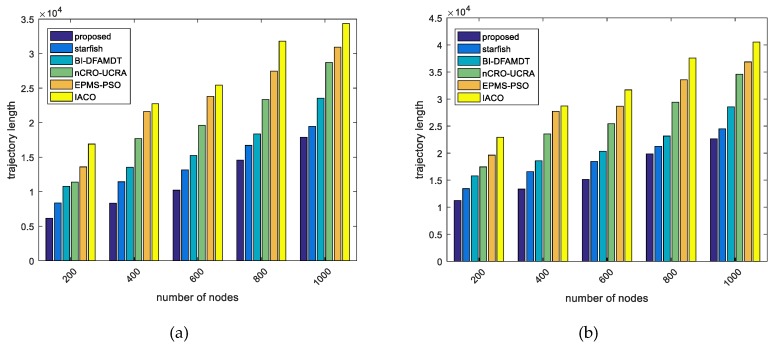
Comparison of the proposed scheme with existing algorithms versus trajectory length travelled by MS: (**a**) Simulation graph for scenario 1; and, (**b**) Simulation graph for scenario 2.

**Figure 15:**
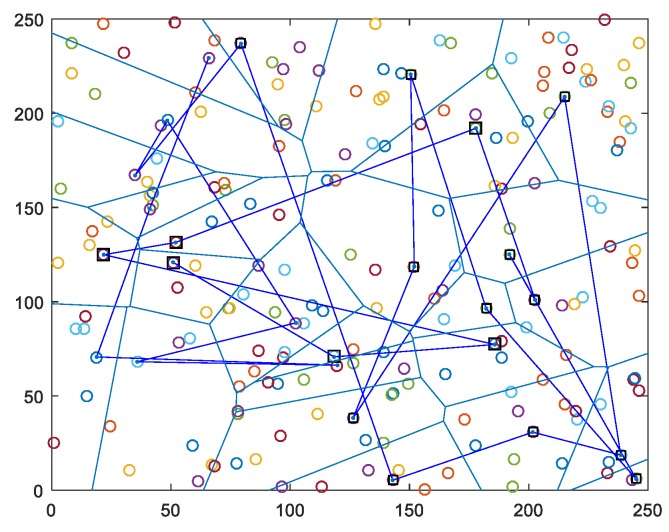
Simulation of the mobile sink trajectory.

**Figure 16:**
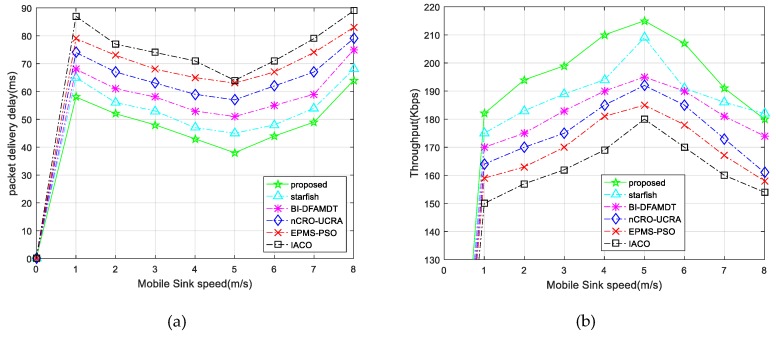
Comparison of the proposed scheme with existing algorithms versus packet delivery delay and throughput: (**a**) Packet delivery delay; and, (**b**) Throughput.

**Figure 17:**
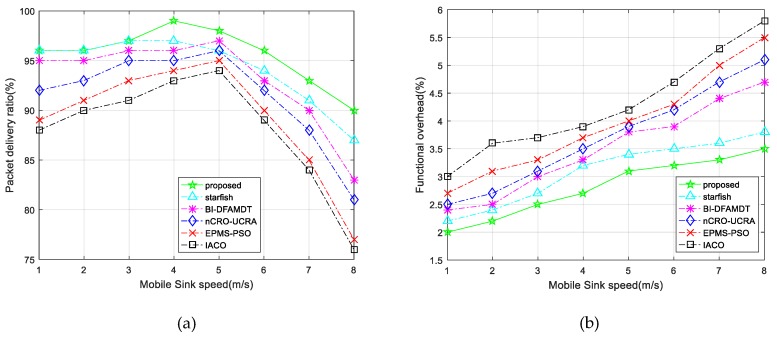
Comparison of the proposed scheme with existing algorithms versus packet delivery ratio and functional overhead: (**a**) Packet delivery ratio; and, (**b**) Functional overhead.

**Figure 18:**
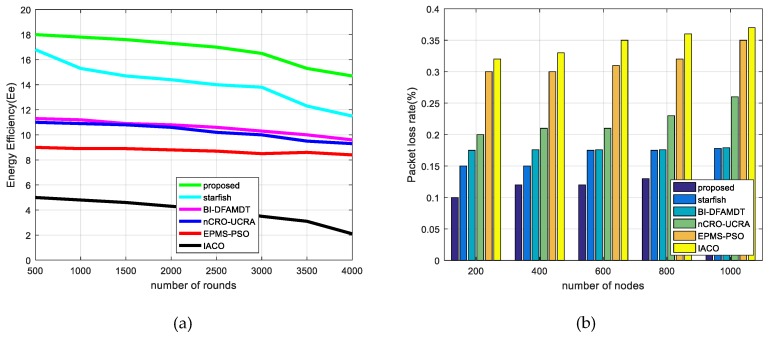
Comparison of the proposed scheme with existing algorithms versus energy efficiency and packet loss rate results: (**a**) Energy efficiency; and, (**b**) Packet loss rate.

**Figure 19:**
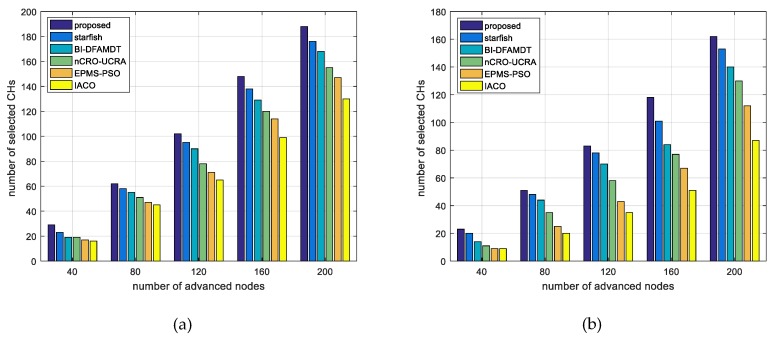
Comparison of the proposed scheme with existing algorithms versus number of selected CHs: (**a**) Simulation graph for scenario 1; and, (**b**) Simulation graph for scenario 2.

Our proposal has generated the minimum travelling path among the existing algorithms due to the fact that the proposed energy-delay and the transmission aware routing protocol is able to determine the best routes for each period. Thanks to the single-hop communication of MS with CH, and visiting the CHs in their transmission range, the trajectory length is shortened. For instance, as illustrated in Figure 14a,b, the proposed algorithm achives the best results almost 0.6 × $10^{4}$ and 1.15 × $10^{4}$ path lengths for 200 nodes in scenario 1 and scenario 2, respectively. However, as the number of nodes increases, the travelling length extends in both scenarios. Additionally, Figure 14b depicts that the trajectory lengths for all of the algorithms are extremely higher values than that of presented in Figure 14a, because of the number of travelling and delivering packet hops in the large- size networks. We ran the proposed algorithm for 200 nodes in scenario 1 and obtained the best trajectory that was travelled by MS, as seen in Figure 15. We do not present the simulation graph of the mobile sink trajectory for scenario 2 to the readers, because the network graph is found as very complex visulation. Actually, we consider that presenting Figure 15 is sufficient in understanding the network model and the proposed methodology of our proposal combined scheme.

v. Packet delivery delay: This is the latency time to deliver data from the source node to the destination node (MS). The lower value means it gives better performance. As shown in Figure 16a, Packet delivery delay decreases in all of the algorithms as the mobile sink speed is about 5 m/s. It occurs, owing to the fact that the sink motility significantly minimizes the hop number and the distance of the sent data packets to the BS. However, higher mobile sink speed leads to obtaining sink possition information. We note that the proposed scheme demonstrates the lowest packet delivery delay among the algorithms compared, as it is able to discover the shortest trajectories.
vi. Throughput: This performance criterion means how accurately the data is transmitted and how accurate the data is delivered from the first bit to the last bit. We have assessed the performances of the algorithm for varying mobile sink speeds from 1 m/s to 8 m/s. The results clearly show that throughput increases with the mobile sink speed up to 5 m/s for all algorithms, as plotted in Figure 16b. However, once the speed of the mobile sink increases more quickly, the throughput value decreases, owing to the fact that MS trajectories change rapidly. The throughput for our proposed algorithm is better than those of other protocols due to the best MS communication with CHs. In this way, MS could gather more accurate data from CHs and deliver it to the BS.
vii. Packet delivery rate: This metric is the ratio of the number of received packets at the mobile sink to the send packets by source nodes. The higher value means that it gives better performance. As illustrated in Figure 17a, the proposed protocol demonstrates the higher packet delivery ratio of 99% with increasing sink speed, especially between 4 and 5 m/s, and this ratio sharply decreases after the speed of the mobile sink increases. This performance validates the benefits of using appropriate mobile sink speed in the network.
viii. Functional overhead: By this performance metric, the number of data packets that is received by the MS is computed. As shown in Figure 17b, the functional overhead of the proposed system is the lowest among the other presented algorithms. This originated because of the clustering of the network and routing to BS with best CH selection in the clusters. This protocol guarantees the single hop communication with BS. On the other hand, it can be observed that functional overhead increases fairly while mobile sink speed increases.
ix. Energy efficiency ($E_{e})$: This metric is used for a performance measurement that observes the network life time. It is calculated via assessing the ratio of the total number of transmitted packets to the BS and the overall network’s energy consumption during the simulation rounds. The formula of energy efficiency ratio $E_{e}$ is presented in Equation (17). We have executed the presented algorithms by simulations according to the energy efficiency during the round iterations. Figure 18a demonstrates that the performance results of the IACO, PSO, nCRO-UCRA, BI-DFAMDT, starfish algorithm, and our proposal scheme are given as the ordered level from the least to most energy efficient level. Although it has partially disrupted after the 3100th round, it has generally demonstrated stable energy efficiency. The proposed algorithm expends less energy thanks to the balanced CH selection algorithm and energy aware routing mechanism in the heterogeneous network.
  (17) $$E_{e}=\frac{\sum The\;number\;of\;packets\;send\;to\;the\;BS}{\sum Total\;energy\;consumption}$$
x. Packet loss rate (PLR): The goal of using this metric, calculated by a formula given in Equation (18), is informing the rate of packet losses due to the functionality of the implemented protocol. We have tested the worked algorithms in terms of packet loss rate versus the number of nodes.
  (18) $$PLR=1-\frac{\sum The\;number\;of\;packets\;received\;by\;the\;BS}{\sum The\;number\;of\;packets\;send\;to\;the\;BS}$$
  As seen in Figure 18b, our proposed algorithm produces the least packet loss rate among the algorithms. As the number of nodes increases in the network, the packet loss rate also increases due to the events of packet collisions and uncorrect packet deliverations.
xi. Number of selected CHs and advanced energy usage rate (%): We also evaluate the advanced energy usage rate as the percentage in the network. We formulate this ratio with the abbreviated name AEUR, as given in Equation (19). Normally, CHs are selected from normal nodes because there is no heterogeneity in other algorithms. However, we used the other algorithms for the comparison terms to be equal. Thus, we distributed the same number of advanced nodes to the network area as our algorithm. We have calculated the number of selected CHs using the algorithms varying from 40 to 200 advanced nodes for scenario 1 (see Figure 19a) and scenario 2 (see Figure 19b). The higher this score, the more performance on behalf of the energy of the advanced nodes is proportionally used in the network. If more CH is selected than the existing advanced nodes, the energy of the advanced nodes is not wasted. As seen in Figure 19a,b, when using our proposed algorithm, the number of selected CHs is higher for both scenarios when compared to other algorithms. However, in scenario 2, the number of selected CHs decreases because of the distance factor and the uncontrolable effect of selecting CHs (see Figure 19b). Table 7 and Table 8 present the advanced energy usage results for scenario 1 and scenario 2, respectively. According to the results, it can be said that our proposal combined scheme has achieved 88.1 and 72.8 AEUR for scenario 1 and scenario 2, respectively.
  (19) $$\begin{array}{c} dvanced\;energy\;usage\;rate\left(\%\right)=AEUR\\ \;=\sum \frac{The\;number\;of\;selected\;CHs}{The\;total\;number\;of\;advanced\;nodes}\times 100 \end{array}$$

## 6. Conclusions and Future Work 

In this study, we offer several solutions for optimization problems, including multi-hop in CH selection and orientation in clustering. For this purpose, we propose two algorithms in this work. First, we present a bacterial interaction and foraging-based CH selection method. The main purpose of this protocol is to create the best set of CHs, when considering energy balance, node degree and energy level, distance, and cost function parameters with an integrated manner. Thanks to the designed CH selection algorithm, MS can collect data by single-hop as soon as it approaches within the transmission boundary range of CH. Hence, we get rid of the multi-hop challenge by this algorithm. Secondly, we propose a routing algorithm that can work dynamically with energy and transmission boundary range awareness, using the $\left[CH_{1\;},\;CH_{2\;},\ldots ,CH_{n}\right]$ set that was obtained as a result of the first algorithm. Thus, MS learns the best trajectory that was obtained by visiting the all of the CHs in the most advantageous way. The MS detects CHs in the coverage areas, called intersection point, representing the path in our algorithm, and it adds this path to the route savings. The proposed scheme was simulated and compared with the IACO, PSO, nCRO-UCRA, BI-DFAMDT, and starfish algorithm by varying number of nodes, network sizes, and MS speeds. This study shows superiority over the competitive studies with respect to energy efficiency, packet loss rate, number of alive nodes in the network, total remaining energy of the network, number of packets received by BS, trajectory length travelled by MS, packet delivery delay, throughput, packet delivery rate, advance energy usage rate, and functional overhead performace metrics; this claim can be observed from the simulation results that were obtained from the Matlab 2016b software. It can be said that our study is more adaptive and applicable in real WSN scenarios. However, future work will be focused on fault tolerance and detection or time complexity issues that are not addressed in the designed network. We plan to develop new methods to overcome these problems. 

## Figures and Tables

**Table 1:** Similarity between bacteria life and wireless sensor network (WSN).

Bacteria Foraging Inspiration	WSN
Bacteria	Sensor nodes
Food density	Objective function
The density of nutrients in the region is not known by bacteria.	The nodes do not know the cost function in advance.
Bacteria can detect the density of food at their location.	The nodes can only detect changes in the values of the cost function.
Bacteria have a limited detection ability (which determines the local error signal) that they cannot assess the intensity, they are only aware of the sign.	When moving nodes, they are aware that only the cost function is increasing or decreasing.
When the nutrient density increases, the bacteria will work in this direction. Otherwise, it is affected by confusion and the bacteria performs tumble movement.	An i node follows the direction of its neighbors in the right direction. Otherwise, if none of the neighbors (including i) are in the correct direction, then the node i will change direction.

**Table 2:** The some notations used in the algorithms.

Notation Symbols	Notation Descriptions
$Problem_{size}$	Problem space
$Step_{size}$	Random direction vector with the same number of dimensions.
$sn_{num}$	The number of bacterial cells that represents sensor node in our algorithm.
$E_{max}$	Initial energy of a sensor node
$L_{max}$	Maximum sensor node load
$E_{remain}$	Remaining energy of a sensor node
$L_{node}$	Load of a sensor node
$D_{node}$	Node degree of a CH
$D_{dist}$	Euclidean distance between a sensor node and an CH within its range
R	Transmission range of a sensor node
∝	Used for trade-off between residual energy and node load
β	Used for trade-off between load distribution and energy consumption
δ	Using for control the number of CHs
$N_{neigh}$	Neighbors of a sensor node that are within its transmission range
$N_{c}$	The number of chemotaxis steps.
$N_{s}$	The number of swim steps for a given bacterial cell
P	The number of dimensions on a given bacterial cells position vector.
$N_{re}$	The number of reproduction steps.
$N_{ed}$	The number of elimination-dispersal steps.
$P_{ed}$	Possibility of elimination and dispersal of a bacterial cell.
*Population*	Bacterial population
$d_{attr}$	Attraction coefficient 1
$w_{attr}$	Attraction coefficient 2
$h_{repel}$	The height of the repellant effect (magnitude of its effect)
$w_{repel}$	Measuring of the width of the bacterial repellant effect

**Table 3:** The segment descriptions of the packet format.

Segment	Description
*id*	Unique definition given by a node
*node(i)Id*	source node *id*
*CHiD*	CH destination *id*
*Eremain*	Remaining energy of a sensor node
*Emax*	Maximum energy of sensor node
*Lnode*	Load of a sensor node
*Lmax*	Maximum oad of sensor node
*DATA*	Transferred data

**Table 4:** The segment descriptions of the routing packet format.

Segment	Description
*id*	Unique definition given by a CH node
*CH(i)Id*	source CH node *id*
*MSiD*	MS destination *id*
*RSSI_CH(i)_MS*	RSSI distance between $CH_{i}$ and MS
*CHofIP(i)*	CH intersection point
*limitArea*	Fields assigned by trajectory edges
*M_CDA*	Collected and aggregated data by CH

**Table 5:** List of the network parameters.

Parameters	Value
Network size	250 × 250 $m^{2}$ and 500 × 500 $m^{2}$
Number of sensor nodes	200 to 1000
Number of advanced nodes	40–200
Initial energy of normal nodes	1 J
Initial energy of advanced nodes	2 J
Data packet size (l)	5000 bits
Transmission range (R)	60 m
Sensor node deployment	Uniformly random
Base station (BS) position	(0,0)
Mobile sink speed	1 m/s to 8 m/s
$e_{fs}$	$10\mathrm{nJ}/\mathrm{bit}/m^{2}$
$e_{amp}$	$0.0015\mathrm{pJ}/\mathrm{bit}/m^{4}$
$E_{elec}$	60nJ/bit
$E_{DA}$	5nJ/bit/signal
$d_{0}$	70 m
$T_{p}$	4000 bits
p	0.5
k	2500
A	50
$L_{cost}$	50
Simulation time (T)	0.5 h

**Table 6:** List of the other parameters used in the proposed algorithms.

Parameters	Value	Parameters	Value
$Problem_{size}$	2	$N_{ed}$	2
$sn_{num}$	50–100	$P_{ed}$	0.2
$Step_{size}$	0.1	$d_{attr}$	0.1
$N_{s}$	4	$w_{attr}$	0.2
$E_{max}$	2 J	$h_{repel}$	0.1
$L_{max}$	5	$w_{repel}$	10
$D_{node}$	4	R	50–80 m
$N_{c}$	50	∝	0.6
$N_{re}$	4	β	0.4

**Table 7:** Advanced energy usage results for scenario 1.

The Number of Total Advanced Nodes	The Number of Selected CHs					
	Proposed	Starfish	BI-DFAMDT	nCRO-UCRA	EPMS-PSO	IACO
40	29	23	19	19	17	16
80	62	58	55	51	47	45
120	102	95	90	78	71	65
160	148	138	129	120	114	99
200	188	176	168	155	147	130
Total	529	490	461	423	396	355
AEUR(%)	88.1	81.6	76.8	70.5	66.0	59.1

**Table 8:** Advanced energy usage results for scenario 2.

The Number of Total Advanced Nodes	The Number of Selected CHs					
	Proposed	Starfish	BI-DFAMDT	nCRO-UCRA	EPMS-PSO	IACO
40	23	20	14	11	9	9
80	51	48	44	35	25	20
120	83	78	70	58	43	35
160	118	101	84	77	67	51
200	162	153	140	130	112	87
Total	437	400	352	311	256	202
AEUR(%)	72.8	66.6	58.6	51.8	42.6	33.6
